# Immunohistochemical Breast Cancer Profiling Among Iraqi Women: Molecular Subtype Classification, Clinicopathology Associations, and Treatment‐Decision Making Tools: A Cross‐Sectional Study

**DOI:** 10.1002/hsr2.70553

**Published:** 2025-05-19

**Authors:** Alaa Salah Jumaah, Roaa Ali Shaker, Zainab Al‐Ali, Kaswer Musa Jaafar Altoriah, Aseel Al‐Quzweni, Salam Salah Jumaah, Akeel Abed Yasseen, Katherine Ann McAllister, Haider J. Al Shiblawi

**Affiliations:** ^1^ Department of Pathology and Forensic Medicine, Faculty of Medicine University of Kufa Kufa Iraq; ^2^ Al‐Kufa Board Training center, Iraqi Board for medical specialization Kufa Iraq; ^3^ Department of Pathology and Forensic Medicine, Faculty of Medicine University of Kerbala Kerbala Iraq; ^4^ Sadder teaching hospital Najaf Governorate Kufa Iraq; ^5^ Euphrates cancer hospital Kufa Iraq; ^6^ School of Biomedical Sciences Ulster University Coleraine Northern Ireland UK

**Keywords:** breast cancer, clinicopathology, immunohistochemistry, molecular subtyping, survival

## Abstract

**Background and Aims:**

Breast cancer is the most common cause of female cancer‐related death in Iraq. This study aimed to classify breast cancer molecular subtypes in the Iraqi population, and investigate the association with clinicopathology parameters, and predict survival outcomes.

**Methods:**

This cross‐sectional study collected breast cancer samples that included: tumor size, grade, lymph node involvement, and LVI. Cases were stained for estrogen (ER) and progesterone (PR) receptors, HER2, and Ki‐67 for IHC subtyping. HER2 score 3 cases were further evaluated by SISH. Molecular profile classification used the St Gallen consensus. The tumor profiles were modelled with treatment combinations using the PREDICT decision making tool of up to 10‐year OS outcomes; > 5% differences were deemed clinically relevant.

**Results:**

The mean age of patients was 50.16 ± 12.28 years. ER and PR positivity was high (81.8% and 73.7%) relative to HER2 (20.8%). Significant clinicopathology associations occurred between ER expression and tumor type and grade (*p* = 0.001, 0.027); HER2 with histology (*p* = 0.044). Ki‐67 high expression (26.8%) was associated with LVI (*p* = 0.006). Molecular classification (IHC subtypes) identified Luminal A (Luminal‐A‐like) tumors in most cases (61.7%), followed by Luminal B (Luminal‐B‐like) (20.0%), HER2 (9.5%) and basal‐like (triple negative breast cancer (TNBC)) at 8.7%. By selecting the right treatment adjuvant to tumor profile, PREDICT modelling estimated that most post‐surgery patients (85.7%, ER^+^; 100%, ER^‐^) would have clinically relevant overall survival (OS) benefit.

**Conclusion:**

St Gallen molecular characterisation of breast tumors is critical for refining triage of healthcare patients in Iraq. Molecular classification using IHC subtypes identified a high prevalence of favorable Luminal‐A‐like type, and the lowest worldwide rates of poor prognostic TNBC cancer. The use of immunohistochemistry‐based cancer subtyping is further strengthened in clinical practice with online prognostication tools that assist the treatment selection process.

## 1 Introduction

Breast cancer is the most frequently diagnosed female cancer and is a serious global health problem [1]. GLOBOCON statistics reported approximately 2.3 million new breast cancer cases and 685,000 deaths worldwide in 2020 [2]. The GLOBACAN age‐standardized incidence and mortality rates of breast cancer are 47.8 and 13.6 per 100,000 population worldwide in 2020. Epidemiology of breast cancer is variable among countries. The age‐standardized incidence ranges from 35.8 (Iran) to 112.3 (Belgium) per 100,000 cases per population, while the age‐standardized mortality ranges from a low of 6.6 in Fiji to 41 per 100,000 of South Koreans. Breast cancer is also the leading cause of cancer‐related death among Iraqi women. Over a ten‐year period in Iraq, cases increased from 26.6/100,000 to 31.5/100,000 [3]; while the age‐standardized incidence and mortality of breast cancer are higher than worldwide averages, with estimates of 54 and 23.3 per 100, 000 cases, respectively. There is currently an urgent need for innovative strategies to improve the outlook of patients with breast cancer in this region.

Breast cancer is a heterogeneous and phenotypically diverse disease, and personalized medicine is the best strategy for improving treatment care pathway. This involves laboratory classification of cancer subtypes and, ultimately, assigning the best treatment regimen based on the tumor molecular profile and clinicopathology characteristics. Approximately 80% and 65% of breast cancers express the estrogen receptor (ER) and progesterone receptor (PR); 20% of tumors are also HER2 positive. ER status is an important prognostic and predictive factor for endocrine responsiveness in breast cancer [4]. The immunohistochemical analysis of HER2 with estrogen receptor (ER) and progesterone receptor (PR) is recommended for routine clinical practice in the management of breast cancer. Ki‐67 is additionally used as proliferative biomarker of breast cancer and is a known indicator of prognosis and outcome. Breast cancer represents a significant health burden in Iraq, yet existing data on estrogen receptor (ER), progesterone receptor (PR), and human epidermal growth factor receptor 2 (HER2) status are limited. Two studies, encompassing 570 [5] and 486 [6] female Iraqi patients, respectively, have investigated these crucial prognostic markers.

The St. Gallen Group provide expert guidelines to support risk stratification and treatment planning of patients with specific breast cancer subtypes [7]. Breast cancer is classified into four intrinsic subtypes: HER2 positive, Luminal‐A, Luminal‐B, and basal‐like [7, 8, 9]. The molecular subtypes of breast cancer have different clinical and prognostic features that may be exploited when selecting treatments. For example, patients with aggressive HER2 positive breast cancers have a poor outlook with traditional chemotherapy. However, 1 year of adjuvant trastuzumab treatment (HER2 inhibitor) can significantly improve OS outcomes of patients with HER2 positive early breast cancer [10, 11]. The prevalence of molecular subtypes worldwide is also variable. This highlights the importance of tumor classification studies in specific ethnic groups to inform treatment care pathways.

The use of immunohistochemistry‐based cancer subtyping is further strengthened with prognostication tools that assist the treatment selection stage. For example, the web‐based prognostication tool PREDICT (http://www.predict.nhs.uk) is freely available and estimates the probability of survival according to systemic treatment choices for individual patients with breast cancer [12, 13]. Such tools provide a simple and economically feasible option in resource limited countries [14], and are ideal to augment the Iraq oncology healthcare system.

The primary goal of this study was breast tumor‐profiling (ER, PR, HER2, Ki‐67) to determine the distribution of molecular subtypes, and clinicopathology characterization across a large cross section of patients with breast cancer attending a cancer center in Iraq. The study also piloted use of the online PREDICT tool on a subset of the tumor‐profiled patient cohort to investigate impact of treatment choices on survival.

## 2 Materials and Methods

### 2.1 Study Ethical Approval and Patient Consent Information

This is a cross‐sectional retrospective analytic study. Ethical approval was obtained from the local Faculty of Medicine ethics committee (IRB number 950 in 2020). The study was performed in accordance with the 1964 Helsinki declaration and its later amendments. Formal written informed consent was obtained from the study participants, who consented to use of residual formalin fixed and paraffin embedded breast cancer material. The study was conducted in collaboration with medical staff at the Euphrates Cancer Hospital, Kufa, Najaf, province of Iraq. Newly diagnosed patients with invasive breast cancer at the hospital received first diagnosis. None of the patients received any type of treatment before biopsy/surgery. Tumor specimens collected for routine hospital diagnosis consisted of mainly of tru‐cut biopsies (*n* = 937), followed by surgical mastectomy (*n* = 274), and a small number of excisional biopsies (*n* = 16). Immunohistochemistry analysis of residual formalin fixed breast cancer specimens was performed by the Department of Pathology and Forensic Medicine, University of Kufa in Iraq. All cases were re‐evaluated by two expert pathologists for diagnosis, staging, grading and immunohistochemically study. Detailed patient data was collected that included: age, histopathologic type, grade, lymph nodes involvement, tumor size, grade, stage and lymphovascular invasion (LVI).

### 2.2 Immunohistochemistry Protocol

All immunohistochemical staining was performed using the DAKO automatic Autostainer Link 48. Five‐micron sections were obtained from paraffin blocks containing each breast cancer case and placed on positive charge slides (DAKO Company) for deparaffinization. The antigen retrieval heat method gradually increased the temperature to 95°C for 20 min, reducing gradually, using the automatic Dako PT Link. The staining procedure used EnVision Flex +, High PH (link) staining kits and ready to use primary antibodies provided by DAKO (on the Dako Autostainer Link 48).

The Flex+ mouse procedure provided signal amplification with the EnVision FLEX visualization system. First slides were rinsed in peroxidase wash buffer, then enzymatically pretreated for 15 min. Slides were rinsed with peroxidase wash buffer. The endogenous peroxidase was blocked using endogenous enzyme block SM801 – Flex peroxidase block for 5 min. Slides were rinsed with peroxidase wash buffer before adding primary antibody (30 min) and rinsing with peroxidase wash buffer. The secondary reagent, SM 804 _ Flex + mouse (Linker) was added for 15 min and rinsed with peroxidase wash buffer. The labelled polymer SM 802 Flex/HRP was added for 20 min, then rinsed with peroxidase wash buffer for 5 min. Flex DAB chromogen was added for 10 min before rinsing. Slides were haematoxylin counter stained (SM 806 Flex) for 5 min, then rinsed with distilled water. Slides were rinsed with peroxidase wash buffer for 5 min and rinsed with distilled water.

### 2.3 Immunohistochemistry Appraisal of Tumors and Molecular Subtyping

Tumors specimen were analyzed in accordance with the 2010 American Society of Clinical Oncology/College of American Pathologists (ASCO/CAP) histopathological consensus guidelines for estrogen (ER) and progesterone (PR) receptor status [15] and reported using the Allred scoring system and the CAP biomarker reporting protocols [16]. Tumor score 2+ for HER2 status were evaluated by SISH to confirm its HER2 condition. (ER, PR, and HER2 reporting are described in the following subsections). Tumor grading was assessed according to the Elston‐Ellis modification of the Scarff‐Bloom‐Richardson grading system, and the World Health Organisation Classification of Tumors Guidelines [17]. Tumor staging was estimated in accordance with the American Joint Committee on Cancer (AJCC), version 8 Guidelines [18]. Ki‐67 expression was estimated using MIB1 antibody testing and recommendations from the International Ki‐67 in Breast Cancer working group [19]. The breast carcinoma molecular subtypes were determined according to the 2013 St. Gallen expert panel consensus [7] based on immunohistochemistry reporting for estrogen progesterone, Ki67 and HER‐2:

1. Luminal A (Luminal‐A‐like): ER + , PR + , HER2− & Low Ki‐67 ( < 14%).
2. Luminal B (Luminal‐B‐like): ER+ and PR+ or PR‐, HER2+ or HER2− & Ki‐67 ≥ 14%.
3. HER2 enriched (HER2 positive): ER − , PR− & HER2+
4. Basal‐like (TNBC): ER − , PR− and HER2−

### 2.4 Estrogen and Progesterone Reporting

Estrogen progesterone reporting was according to Allred scoring system [16]. This score assessed (1) proportion and (2) intensity of stained breast cancer cells.

1. Proportion scoring system: 0 (0% positive), 1 ( < 1 positive), 2 (1% to 10% positive), 3 (11% to 33% positive), 4 (34% to 66% positive), 5 ( ≥ 67% positive).
2. Intensity scores: 0 (none), 1 (weak), 2 (intermediate), and 3 (strong).

The final score was estimated by adding both scores: score 0 and 2 are negative, while score ≥ 3 are positive.

### 2.5 HER2 Reporting

HER2 reporting used ASCO and CAP recommendations for immunohistochemistry [20]. The scoring system:

1. Negative (score 0) “no staining observed or membrane staining which is incomplete and is faint and barely perceptible and within ≤ 10% of tumor cells”,
2. Negative (score 1 + ) “incomplete membrane staining which is faint and barely perceptible within > 10% of tumor cells”,
3. Equivocal (score 2 + ) “weak to moderate complete membrane staining in > 10% of tumor cells or complete staining of membrane that is intense but within ≤ 10% of tumor cells”, and finally
4. Positive score (3 + ) “complete membrane staining which is intense and in > 10% of tumor cells”.

### 2.6 Silver‐Enhanced In Situ Hybridisation Reporting of HER‐2

Equivocal results were further analyzed by the silver‐enhanced in situ hybridization method (SISH), according to ASCO and CAP recommendations (20). The dual probe SISH method used group definitions:

1. (Group 1 = HER2/CEP17 ratio ≥ 2.0; ≥ 4.0 HER2 signals/cell),
2. (Group 2 = HER2/CEP17 ratio ≥ 2.0; < 4.0 HER2 signals/cell),
3. (Group 3 = HER2/CEP17 ratio < 2.0; ≥ 6.0 HER2 signals/cell),
4. (Group 4 = HER2/CEP17 ratio < 2.0; ≥ 4.0 and < 6.0 HER2 signals/cell), and
5. (Group 5 = HER2/CEP17 ratio < 2.0; < 4.0 HER2 signals/cell).

**Figure 1:**
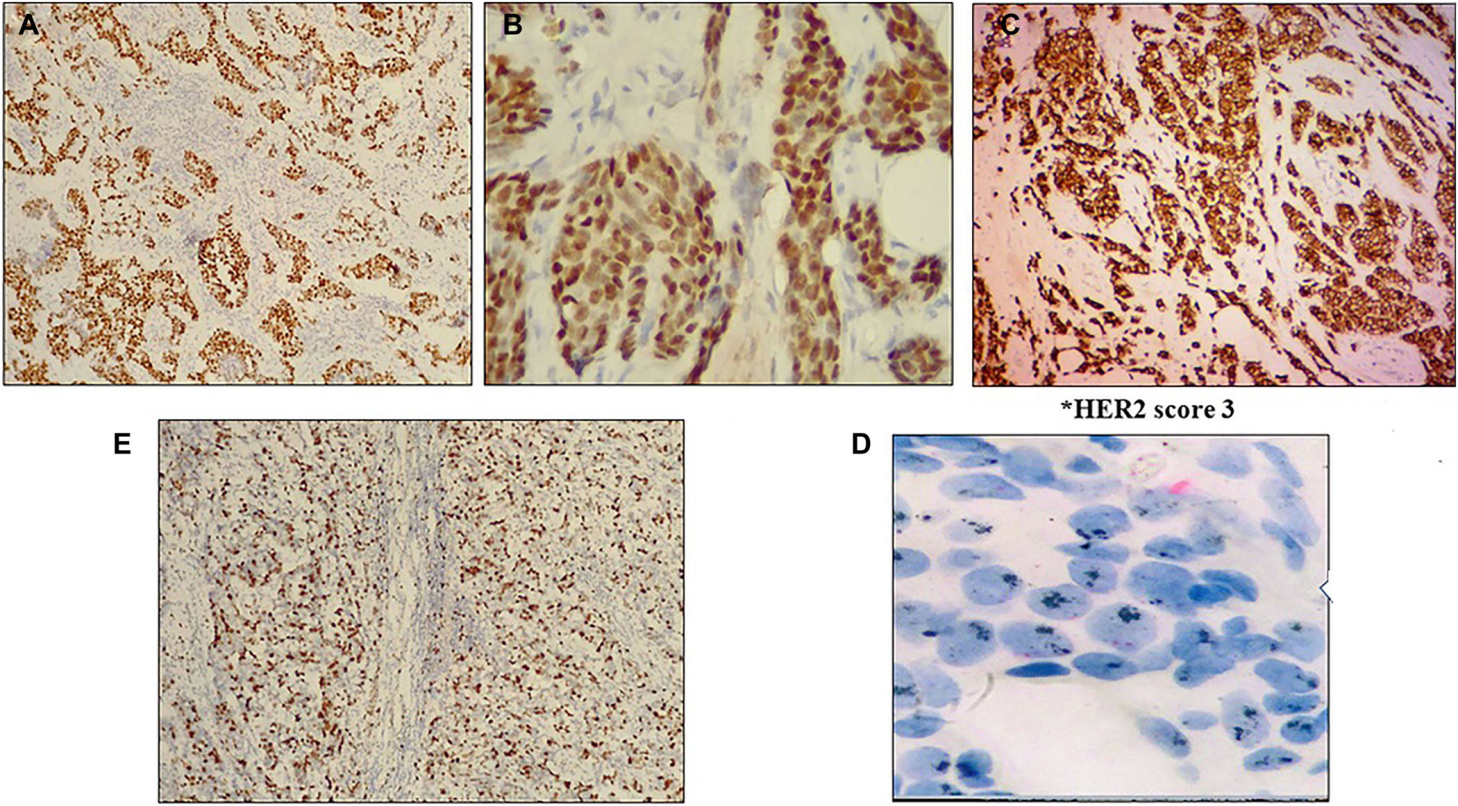
Immunohistochemistry staining of breast cancer tumor markers. (A). Diffuse nuclear staining of ERs in invasive ductal carcinoma (40X); (B) Diffuse nuclear staining of PRs in invasive ductal carcinoma (40X); (C) HER2 positive score 3+ with uniform complete membranous staining (40X); (D) HER2 amplified with presence of HER2 SISH clusters. (60X); (E) High expression of KI67 in breast carcinoma (40X).

Reporting Results of HER2 Testing by In Situ Hybridization (dual‐probe assay):

1. Negative (group 5),
2. Negative (group 2 and concurrent IHC 0‐1+ or 2+ or group 3 and concurrent IHC 0‐1+ or group 4 and concurrent IHC 0‐1+ or 2 + ),
3. Positive (group 2 and concurrent IHC 3+ or group 3 and concurrent IHC 2+ or 3+ or group 4 and concurrent IHC 3 + ),
4. Positive (group 1). Refer to Figure 1D for example of HER2 score 3 staining results.

### 2.7 Application of Decision Making Tools: PREDICT Treatment Modelling

Use of predictive online treatment calculators are encouraged by The American Joint Committee on Cancer (AJCC) and early breast cancer guidelines.

The PREDICT tool was applied for women who have had surgery for early invasive breast cancer to consider adjuvant treatments. Patient data was manually entered into the PREDICT platform (https://breast.predict.nhs.uk/): including patient age, tumour size, tumour grade, number of positive nodes, ER status, HER2/ERRB2 status, Ki‐67 status and mode of detection. Survival estimates in breast cancer patients treated with and without adjuvant therapy (chemotherapy, hormone therapy, trastuzumab and bisphosphonates for post‐menopausal patients) were retrieved.

Exclusion criteria: patients with unknown tumor size, number of positive lymph nodes, differentiation grade or ER status were excluded, since PREDICT does not allow missing values for these variables

Decision making for adjuvant chemotherapy was considered using the Cambridge Breast Unit (UK), which uses the absolute 10‐year survival benefit from chemotherapy to guide decision making for adjuvant chemotherapy as follows:

- < 3% chemotherapy not recommended
- 3–5% chemotherapy discussed as a possible option
- > 5% chemotherapy recommended.

### 2.8 Statistical Analysis

Clinicopathology, demographics, and immunohistochemical results were analyzed using descriptive statistics. The analysis of variance and the Chi‐Square Test were used to examine the association of the ER, PR, HER2, and Ki67 status and Molecular Subtypes (IHC groups/subtypes or IHC grouping) with clinicopathologic prognostic features. Fisher's exact test analysis was also undertaken. PREDICT script was used to calculate the expected 5 and 10 year OS of a selected cohort, separated by ER status, and treatment subgroups based on age, HER2 status, and type of adjuvant systemic therapy. Nonparametric analysis used Wilcoxon matched‐pairs signed rank to compare surgery to adjuvants, and Friedman testing to compare treatment groups with the same number of cases. All tests of significance were 2‐tailed, with a level of significance *p* < 0.05 denoting statistical significance. The raw data were initially recorded on paper data collection forms and subsequently entered into Microsoft Excel 2013 spreadsheets for data cleaning and preliminary analysis. The Statistical package for Social Sciences IBM SPSS Statistics version 26 (Arbuckle, J. L. (2019). Amos (Version 26.0) [Computer Program] Chicago: IBM SPSS), and GraphPad Prism were used for final data analysis.

#### 2.8.1 Ethics and Consent Statement

All procedures performed in the current study were approved by IRB and/or national research ethics committee (IRB 950; February 25th, 2020) in accordance with the 1964 Helsinki declaration and its later amendments. Informed consent was obtained from all individual participants included in the study. All authors have read and approved the final version of the manuscript. The manuscript guarantor (Professor Akeel Yasseen) had full access to all of the data in this study and takes complete responsibility for the integrity of the data and the accuracy of the data analysis.

## 3 Results

### 3.1 Characteristics of Patients with Breast Cancer

A total of 1,227 patients with breast cancer were assessed for immunohistochemistry and clinicopathology as reported in Table 1.

**Table 1:** Patient characteristics and breast tumor parameters.

Patient characteristics	Tumor characteristics (*N* = 1,227)	Frequency, *n* (%)
Histologic type (*N* = 1,227)	IDC^a^	958 (78.1)
	ILC^b^	194 (15.8)
	Other	75 (6.1)
Number of cases stained for tumor markers (*N* = 1,227)	ER^c^ positive	1,004 (81.8)
	ER negative	223 (18.2)
	PR^d^ positive	904 (73.7)
	PR negative	323 (26.3)
	HER2 positive	255 (20.8)
	HER2 negative	972 (79.2)
	Ki‐67 high/low	329/898 (26.8/73.2)
Grade (*N* = 495)	I	40 (8.1)
	II	344 (69.5)
	III	111(22.4)
Tumor size (*N* = 290)	T1	37 (12.8)
	T2	148 (51.0)
	T3	76 (26.2).
	T4	29 (10.0)
Lymph nodes (*N* = 309)	N0	83 (26.9)
	N1	97 (31.4)
	N2	75 (24.3)
	N3	54 (17.5)
LVI^e^ (*N* = 334)	No	273 (81.7)
	Yes	61 (18.3)

^a^ IDC, invasive ductal carcinoma.

^b^ ILC, invasive lobular carcinoma.

^c^ ER, estrogen receptor.

^d^ PR, progesterone receptor.

^e^ LVI, lymph vascular space invasion.

The average age of all patients at the time of diagnosis was 50.16 ± 12.28 years. Histology reported mainly invasive ductal carcinoma (*n* = 958, 78.1%), a few patients had invasive lobular carcinoma (*n* = 194, 15.8%), the remainder had other histological types (*n* = 75, 6.1%). All tumor specimens (*N* = 1,227) were stained for four IHC markers, including ER, PR, HER2, and Ki‐67. Most histological breast cancers showed high percentage of receptor positivity for estrogen and progesterone (81.8% and 73.7%) as shown in Figure 1A and B, and lower HER2 (Figure 1C) positivity (20.8%). Cases with HER2 score 2 proceeded to the SISH technique for further evaluation as shown in Figure 1D. Ki67 staining was ‘high’ in 26.8% and ‘low’ in 73.2% (Figure 1E).

Clinicopathology information was available for subsets of the patient study population: tumor grade subset (*n* = 495), tumor size subset (*n* = 290), lymph nodes subset (*n* = 309), and LVI subset (*n* = 334). Most cancers were grade II (69.5%), sized T2 (51.0%), and around 75% had reached 1–3 lymph nodes. LVI developed in 18.3% of the valid study population.

### 3.2 Stratification of Tumor Markers With Clinicopathology Features

The stratification of tumor makers according to clinicopathology is outlined in Table 2. ER expression showed no significant association (*p*> 0.05) with age, tumor size, lymph node involvement, and LVI parameters. ER expression had significantly association with histological tumor type (*p* = < 0.001) and tumor grade (*p* = 0.02). Higher levels of ER were noted in IDC (62.8%) compared to ILC (14.6%), or other histological types (4.4%). ER positive tumors were mainly grade II (59.4%). There was no significant association (*p*> 0.05) in PR expression with regard to age, tumor size (T), tumor grade, lymph nodes involvement, and LVI. The progesterone receptors had a similar pattern of positive expression as estrogen, in regard to significant association with histologic type (*p* = 0.04).

**Table 2:** Stratification of ER, PR, and HER2, and Ki‐67 to clinicopathology characteristics.

Characteristics (*N*, 1,227)			ER^‐^	ER^+^	PR^‐^	PR^+^	HER2^‐^	HER2^+^	Ki‐67 High	Ki‐67 Low
Mean age (SD) *n* (%)			49.76 (12.79)	50.25 (12.24)	49.76 (12.79)	50.25 (12.24)	50.30 (12.18)	49.06 (13.56)	49.68 (12.34)	50.34 (12.34)
*p* value			0.592		0.500		0.273		0.408	
Histologic type *N* = 1227 *n* (%)	IDC^a^	Count	187	771	255	703	749	209	243	715
		% within histologic type	19.5%	80.5%	26.6%	73.4%	78.2%	21.8%	25.4%	74.6%
		% within marker	83.9%	76.8%	78.9%	77.8%	77.1%	82.0%	73.9%	79.6%
	ILC^b^	Count	15	179	41	153	166	28	64	130
		% within histologic type	7.7%	92.3%	21.1%	78.9%	85.6%	14.4%	33.0%	67.0%
		% within marker	6.7%	17.8%	12.7%	16.9%	17.1%	11.0%	19.5%	14.5%
	Others	Count	21	54	27	48	57	18	22	53
		% within histologic type	28.0%	72.0%	36.0%	64.0%	76.0%	24.0%	29.3%	70.7%
		% within marker	9.4%	5.4%	8.4%	5.3%	5.9%	7.1%	6.7%	5.9%
	Total	Count	223	1004	323	904	972	255	329	898
		% within histologic type	18.2%	81.8%	26.3%	73.7%	79.2%	20.8%	26.8%	73.2%
		% within marker	100.0%	100.0%	100.0%	100.0%	100.0%	100.0%	100.0%	100.0%
*p* value			< 0.001*		0.04*		0.04*		0.08	
Grade *N* = 495 *n* (%)	I	Count	4	36	7	33	30	10	8	32
		% within Grade	10.0%	90.0%	17.5%	82.5%	75.0%	25.0%	20.0%	80.0%
		% within marker	1.8%	3.6%	2.2%	3.7%	3.1%	3.9%	2.4%	3.6%
	II	Count	50	294	75	269	283	61	118	226
		% within Grade	14.5%	85.5%	21.8%	78.2%	82.3%	17.7%	34.3%	65.7%
		% within marker	22.4%	29.3%	23.2%	29.8%	29.1%	23.9%	35.9%	25.2%
	III	Count	27	84	35	76	89	22	32	79
		% within Grade	24.3%	75.7%	31.5%	68.5%	80.2%	19.8%	28.8%	71.2%
		% within marker	12.1%	8.4%	10.8%	8.4%	9.2%	8.6%	9.7%	8.8%
	Total		81	414	117	378	402	93	158	337
			16.4%	83.6%	23.6%	76.4%	81.2%	18.8%	31.9%	68.1%
*p* value			0.02*		0.07		0.51		0.13	
Tumor size (T) *N* = 292 *n* (%)	T1	Count	3	34	5	32	32	5	11	26
		% within T	8.1%	91.9%	13.5%	86.5%	86.5%	13.5%	29.7%	70.3%
		% within marker	7.0%	13.8%	7.4%	14.4%	13.5%	9.4%	11.6%	13.3%
	T2	Count	27	121	39	109	118	30	50	98
		% within T	18.2%	81.8%	26.4%	73.6%	79.7%	20.3%	33.8%	66.2%
		% within marker	62.8%	49.0%	57.4%	49.1%	49.8%	56.6%	52.6%	50.3%
	T3	Count	10	66	18	58	66	10	26	50
		% within T	13.2%	86.8%	23.7%	76.3%	86.8%	13.2%	34.2%	65.8%
		% within marker	23.3%	26.7%	26.5%	26.1%	27.8%	18.9%	27.4%	25.6%
	T4	Count	3	26	6	23	21	8	8	21
		% within T	10.3%	89.7%	20.7%	79.3%	72.4%	27.6%	27.6%	72.4%
		% within marker	7.0%	10.5%	8.8%	10.4%	8.9%	15.1%	8.4%	10.8%
	Total	Count	43	247	68	222	237	53	95	195
		% within T	14.8%	85.2%	23.4%	76.6%	81.7%	18.3%	32.8%	67.2%
		% within marker	100.0%	100.0%	100.0%	100.0%	100.0%	100.0%	100.0%	100.0%
*p* value			0.34		0.41		0.26		0.88	
Lymph nodes, *N* = 309 *n* (%)	N0	Count	19	64	28	55	66	17	28	55
		% within N	22.9%	77.1%	33.7%	66.3%	79.5%	20.5%	33.7%	66.3%
		% within marker	41.3%	24.3%	36.4%	23.7%	26.3%	29.3%	26.7%	27.0%
	N1	Count	12	85	16	81	81	16	31	66
		% within N	12.4%	87.6%	16.5%	83.5%	83.5%	16.5%	32.0%	68.0%
		% within marker	26.1%	32.3%	20.8%	34.9%	32.3%	27.6%	29.5%	32.4%
	N2	Count	9	66	18	57	58	17	23	52
		% within N	12.0%	88.0%	24.0%	76.0%	77.3%	22.7%	30.7%	69.3%
		% within marker	19.6%	25.1%	23.4%	24.6%	23.1%	29.3%	21.9%	25.5%
	N3	Count	6	48	15	39	46	8	23	31
		% within N	11.1%	88.9%	27.8%	72.2%	85.2%	14.8%	42.6%	57.4%
		% within marker	13.0%	18.3%	19.5%	16.8%	18.3%	13.8%	21.9%	15.2%
	Total	Count	46	263	77	232	251	58	105	204
		% within N	14.9%	85.1%	24.9%	75.1%	81.2%	18.8%	34.0%	66.0%
		% within marker	100.0%	100.0%	100.0%	100.0%	100.0%	100.0%	100.0%	100.0%
*p* value			0.12		0.06		0.61		0.50	
LVI^c^ *N* = 334	No	Count	40	233	68	205	225	48	92	181
		% within LVI	14.7%	85.3%	24.9%	75.1%	82.4%	17.6%	33.7%	66.3%
		% within ER	81.6%	81.8%	84.0%	81.0%	82.1%	80.0%	74.2%	86.2%
	Yes	Count	9	52	13	48	49	12	32	29
		% within LVI	14.8%	85.2%	21.3%	78.7%	80.3%	19.7%	52.5%	47.5%
		% within ER	18.4%	18.2%	16.0%	19.0%	17.9%	20.0%	25.8%	13.8%
	Total	Count	49	285	81	253	274	60	124	210
		% within LVI	14.7%	85.3%	24.3%	75.7%	82.0%	18.0%	37.1%	62.9%
		% within ER	100.0%	100.0%	100.0%	100.0%	100.0%	100.0%	100.0%	100.0%
*p* value			0.98		0.55		0.70		0.006*	

^a^ IDC, invasive ductal carcinoma.

^b^ ILC, invasive lobular carcinoma.

^c^ LVI, lymph vascular space invasion.

* significant *p* value (< 0.05); ^‐^ Negative estrogen receptor (ER), progesterone receptor (PR) or HER‐2; ^+^Positive tumor marker expression for estrogen receptor (ER), progesterone receptor (PR), or HER‐2.

HER2 expression was only significantly associated with histology of the tumor. Whereas the proliferation marker Ki‐67 (Figure 1E, Table 2) had significant association with LVI (*p* = 0.006). The expression of Ki‐67 was mainly low (54.2%) in cases without development of LVI.

### 3.3 Distribution of the Breast Cancer Molecular Subtypes (IHC Groups/Subtypes or IHC Grouping)

**Figure 2:**
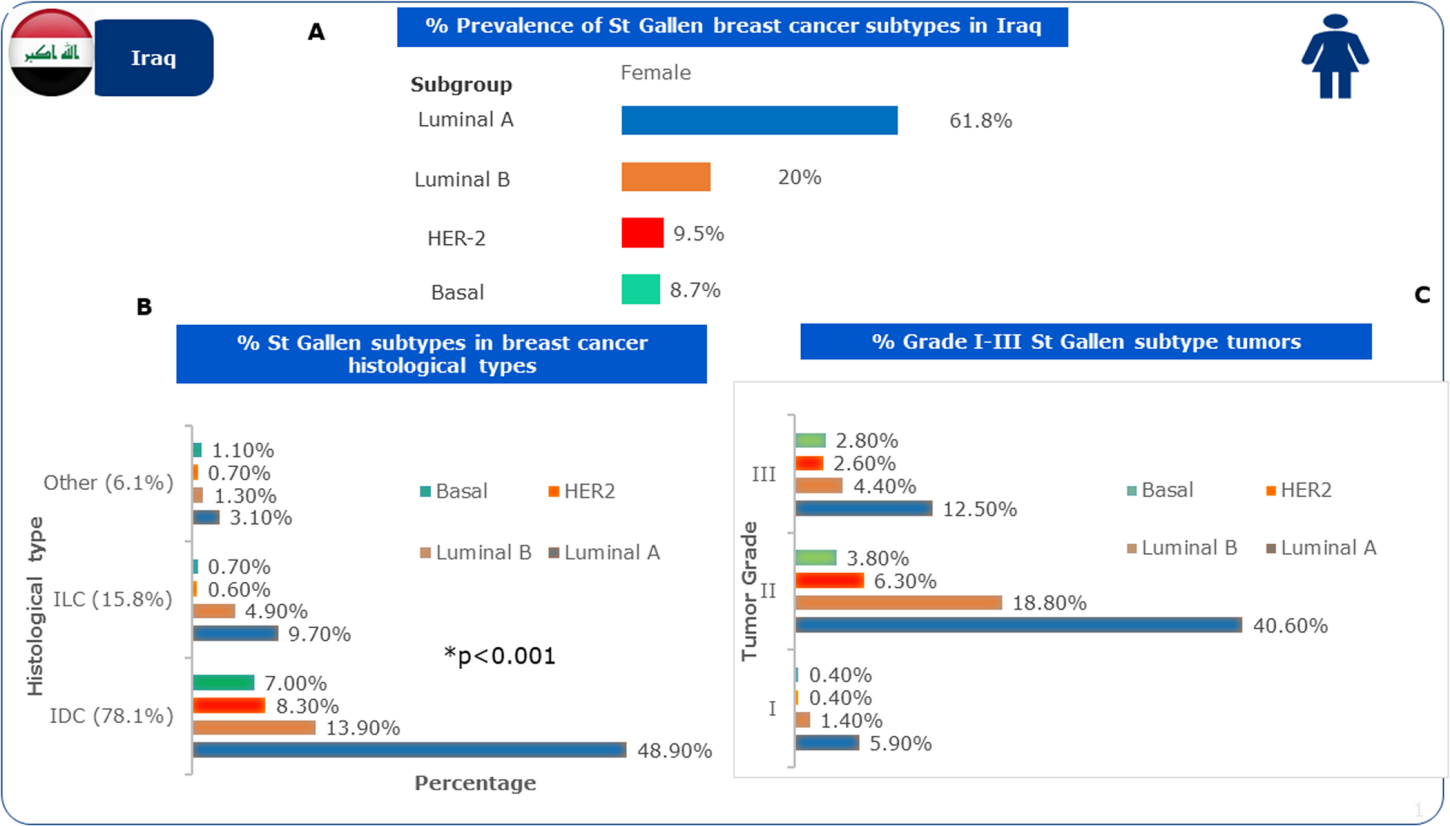
(A) Prevalence of breast cancer molecular subtypes in Iraq; (B) Histological distributions of molecular subtypes; (C) Distribution of molecular subtypes across tumor grades.

The % prevalence of molecular subtypes according to the St Gallen guidelines are shown in Figure 2A and Table 3. Luminal A (Luminal‐A‐like) was the most common type (61.8%), then Luminal B (Luminal‐B‐like) (20.0%), HER2 enriched (HER2 positive) type (9.5%), finally triple negative breast cancer or the basal type profile was noted at 8.7%. There was a significant association between the histologic types and molecular profiles (*p* < 0.001), and nearing significance with tumor grade (*p* = 0.06). Luminal A ((Luminal‐A‐like) and Luminal B (Luminal‐B‐like) tumors had high distribution towards grade II (40.6% and 18.8%) in Figure 2C. There were no other significant associations in regard to tumor size, lymph node involvement, or LVI with the molecular subtypes.

**Table 3:** Clinicopathologic characteristics in association with molecular types (IHC groups/subtypes or IHC grouping) of breast cancers.

Characteristics			Luminal A (Luminal‐A‐like) (*N* = 757, 61.8%)	Luminal B (Luminal‐B‐like) (*N* = 246, 20.0%)	HER2 enriched (HER2 Positive) (*N* = 117, 9.5%)	Basal‐like (Triple negative breast carcinoma‐TNBC)(*N* = 107, 8.7%)	*p* value
Mean age (±SD)			50.59 (11.96)	49.38 ± 12.963	48.51 ± 13.505	50.76 ± 12.081	0.238
Histological types	IDC	Count	600	170	102	86	< 0.001*
		% within histological types	62.6%	17.7%	10.6%	9.0%	
		% within luminal	79.3%	69.1%	87.2%	80.4%	
	ILC	Count	119	60	7	8	
		% within histological types	61.3%	30.9%	3.6%	4.1%	
		% within luminal	15.7%	24.4%	6.0%	7.5%	
	Others	Count	38	16	8	13	
		% within histological types	50.7%	21.3%	10.7%	17.3%	
		% within luminal	5.0%	6.5%	6.8%	12.1%	
	Total	Count	757	246	117	107	
		% within histological types	61.7%	20.0%	9.5%	8.7%	
		% within luminal	100.0%	100.0%	100.0%	100.0%	
Grade	I	Count	29	7	2	2	0.06
		% within Grade	72.5%	17.5%	5.0%	5.0%	
		% within luminal	9.9%	5.7%	4.3%	5.7%	
	II	Count	201	93	31	19	
		% within Grade	58.4%	27.0%	9.0%	5.5%	
		% within luminal	68.8%	76.2%	67.4%	54.3%	
	III	Count	62	22	13	14	
		% within Grade	55.9%	19.8%	11.7%	12.6%	
		% within luminal	21.2%	18.0%	28.3%	40.0%	
	total	Count	292	122	46	35	
		% within Grade	59.0%	24.6%	9.3%	7.1%	
		% within luminal	100.0%	100.0%	100.0%	100.0%	
Tumor size T	T1	Count	24	10	2	1	0.85
		% within T	64.9%	27.0%	5.4%	2.7%	
		% within luminal	14.0%	13.3%	7.7%	5.9%	
	T2	Count	86	35	17	10	
		% within T	58.1%	23.6%	11.5%	6.8%	
		% within Luminal	50.0%	46.7%	65.4%	58.8%	
	T3	Count	43	23	5	5	
		% within T	56.6%	30.3%	6.6%	6.6%	
		% within luminal	25.0%	30.7%	19.2%	29.4%	
	T4	Count	19	7	2	1	
		% within T	65.5%	24.1%	6.9%	3.4%	
		% within luminal	11.0%	9.3%	7.7%	5.9%	
	Total	Count	172	75	26	17	
		% within T	59.3%	25.9%	9.0%	5.9%	
		% within luminal	100.0%	100.0%	100.0%	100.0%	
Lymph nodes (LN)	N0	Count	44	20	10	9	0.27
		% within N	53.0%	24.1%	12.0%	10.8%	
		% within luminal	24.6%	23.8%	34.5%	52.9%	
	N1	Count	62	23	9	3	
		% within N	63.9%	23.7%	9.3%	3.1%	
		% within luminal	34.6%	27.4%	31.0%	17.6%	
	N2	Count	45	21	6	3	
		% within N	60.0%	28.0%	8.0%	4.0%	
		% within luminal	25.1%	25.0%	20.7%	17.6%	
	N3	Count	28	20	4	2	
		% within N	51.9%	37.0%	7.4%	3.7%	
		% within luminal	15.6%	23.8%	13.8%	11.8%	
	Total	Count	179	84	29	17	
		% within N	57.9%	27.2%	9.4%	5.5%	
		% within luminal	100.0%	100.0%	100.0%	100.0%	
LVI	No	Count	157	76	23	17	0.07
		% within LVI	57.5%	27.8%	8.4%	6.2%	
		% within luminal	85.8%	74.5%	76.7%	89.5%	
	Yes	Count	26	26	7	2	
		% within LVI	42.6%	42.6%	11.5%	3.3%	
		% within luminal	14.2%	25.5%	23.3%	10.5%	
	Total	Count	183	102	30	19	
		% within LVI	54.8%	30.5%	9.0%	5.7%	
		% within luminal	100.0%	100.0%	100.0%	100.0%	

*Note:* a, IDC, invasive ductal carcinoma; b, ILC, invasive lobular carcinoma; c, LVI, lymph vascular space invasion. * denotes significant *p* value (< 0.05).

The distribution of breast cancer molecular subtypes in the Iraqi study population was compared to selected populations worldwide [5, 21, 22, 23, 24, 25, 26, 27, 28] reported in Table 4. Breast cancer subtypes are highly variable among different races and ethnicities. The Luminal A (Luminal‐A‐Like) subtype presented highly in North American US Whites at 75.5% but decreases to a low of 24.1% in Southern America (Brazil), Luminal B had highest prevalence in Brazil (39.5%) dropping to lows of 10% in the US (all races); HER2 ranged from 24.1% in the Middle East (Oman) to just 4% in the US. Whereas Basal molecular types vary from 39%% in African Americans to a low of 8.7% in Iraqi patients from the current study.

**Table 4:** Frequency distribution of the breast cancer molecular subtypes in selected global populations.

Location	% Prevalence of molecular subtypes in breast cancer					IHC Surrogate/Classifier	Study size, *N*	Reference
	Luminal A	Luminal B	HER‐2	Basal/Triple negative	Other sub type			
Iraq	61.8%	20.0%	9.5%	8.7%*	—	St Gallen consensus	1,227	This paper
	42.2%	14.6%	11.8%	15.6%	—	Surrogate ER/PC/HER staining	570	[5]
Saudi Arabia	58.5%	14.5%	12.3%	14.8%	—	St Gallen consensus	359	Alnegheimish, Alshatwi [20]
Oman	34.7%	15.9%	24.1%	25.3%	—	Pre Gallen consensus	543	Mehdi, Monem [25]
Korea	65.8% versus 45.0%;	9.0% versus 8.5%	4.1% versus 12.1%	11.2% versus 31.2%	—	NR	302	[26]
US (All)	68%	10%	4%	10%	7%	NR	NR	SEER
US Black (premenopause)	36%	9%	9%	39%		a IHC subtypes: b ER, c PR, HER2, HER1, and cytokeratin 5/6		[21]
US Black (postmenopause)	59%	16%	7%	14%				
US non African	54%			16%				
US White	75.50%	9.80%	4.00%	10.70%	—	ER/PC/HER‐2 status	40,744	[24]
US Black	60.20%	11.40%	6.00%	22.50%	—		6,007	
US Asian Pacific	71.10%	12.30%	6.90%	9.70%	—		4,367	
US Hispanic	68.20%	11.40%	5.70%	14.70%	—		5,694	
Sweden	53%	20%	13%	14%	—	St Gallen consensus	45	[23]
Brazil regions	24.1‐30.8	30.8‐39.5	6.7‐13.5	14‐20.3	9.7‐12.9	Surrogate ER, PR, HER2, Ki‐67, EGFR and CK5/6	5,687	[22]
Africa	40%	26%	10%	23%	—	St Gallen consensus	114	[27]

^a^ IHC, immunohistochemistry,

^b^ ER, estrogen receptor,

^c^ PR, progesterone receptor.

### 3.4 PREDICT Treatment Modelling of Tumor Profiles

A total of 142 breast cancer patients were suitable candidates for modelling treatment survival predictions using immunohistochemistry biomarkers. By matching the right adjuvant to tumor marker profile, PREDICT modelling estimated most patients (85.7%, ER^+^; 100%, ER^‐)^ had clinically relevant OS benefits.

**Figure 3:**
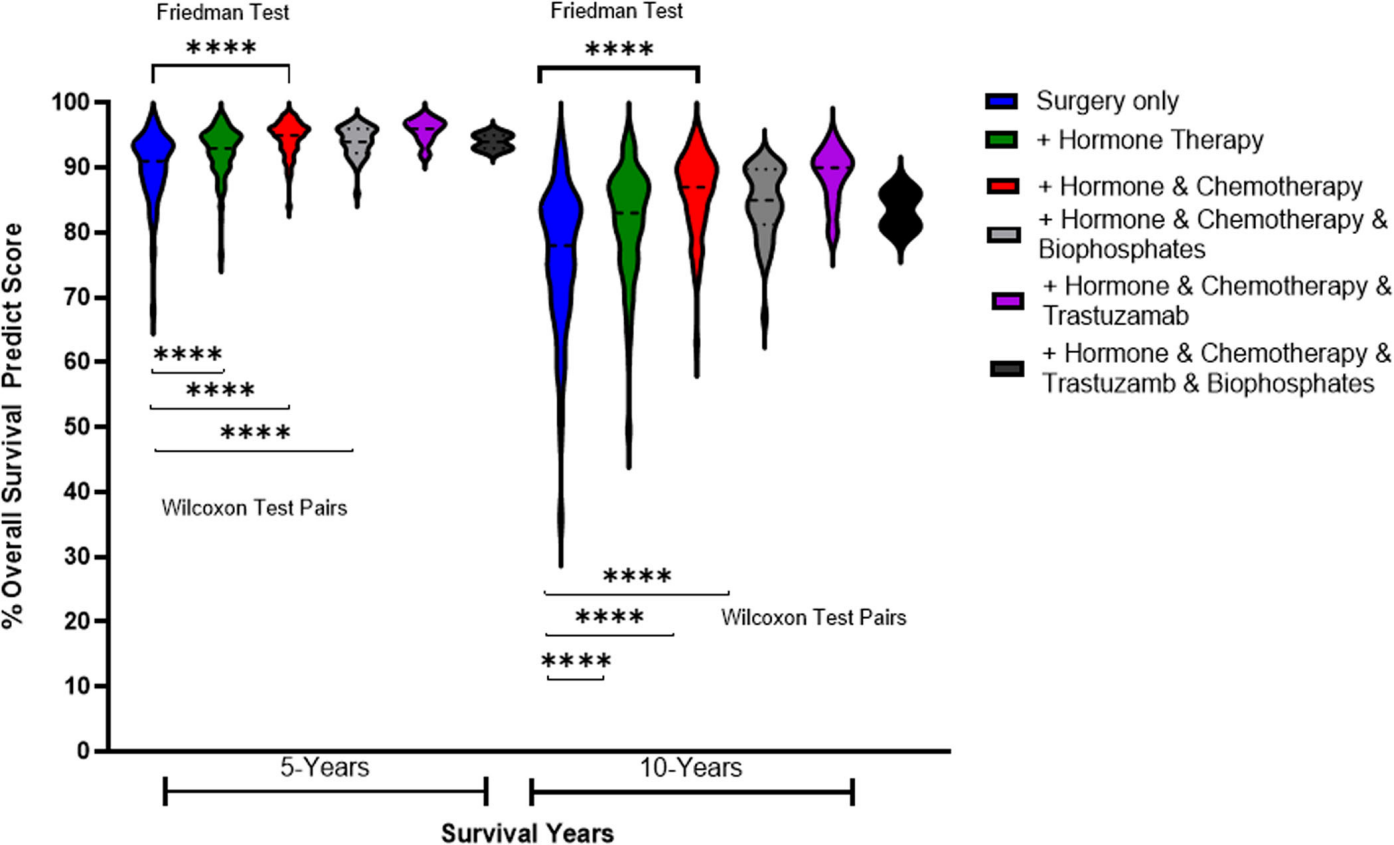
PREDICT Modelling of 5 and 10 Year Survival in ER^+^ Breast Cancers Posttreatment.

In all ER^+^ tumors (Table A1) significantly increasing 5 and 10‐year OS benefits were noted with adjuvant endocrine therapy (*p* < 0.001) combined with chemotherapy (*p* < 0.001) and trastuzumab in HER2^+^ women (*p* = 0.003), compared to surgery alone. The Wilcoxon signed rank test pairs shows that the observed difference were highly significant (*p* < 0.001) between both surgery and hormone therapy and surgery with adjuvant hormone and chemotherapy at 5 and 10 years. The Friedman test showed significant statistically significant difference between the means of these three groups (where the same subjects show up in each group, *n* = 119) (see Figure 3 for ER^+^). The violin plots in Figure 3 illustrates how the distribution of survival in patients is improved by adding adjuvant treatment after surgery, across all added treatments, with the median high to low survival ranges decreasing from 30% (surgery) to just 2% (all adjuvants) at 5 years.

**Figure 4:**
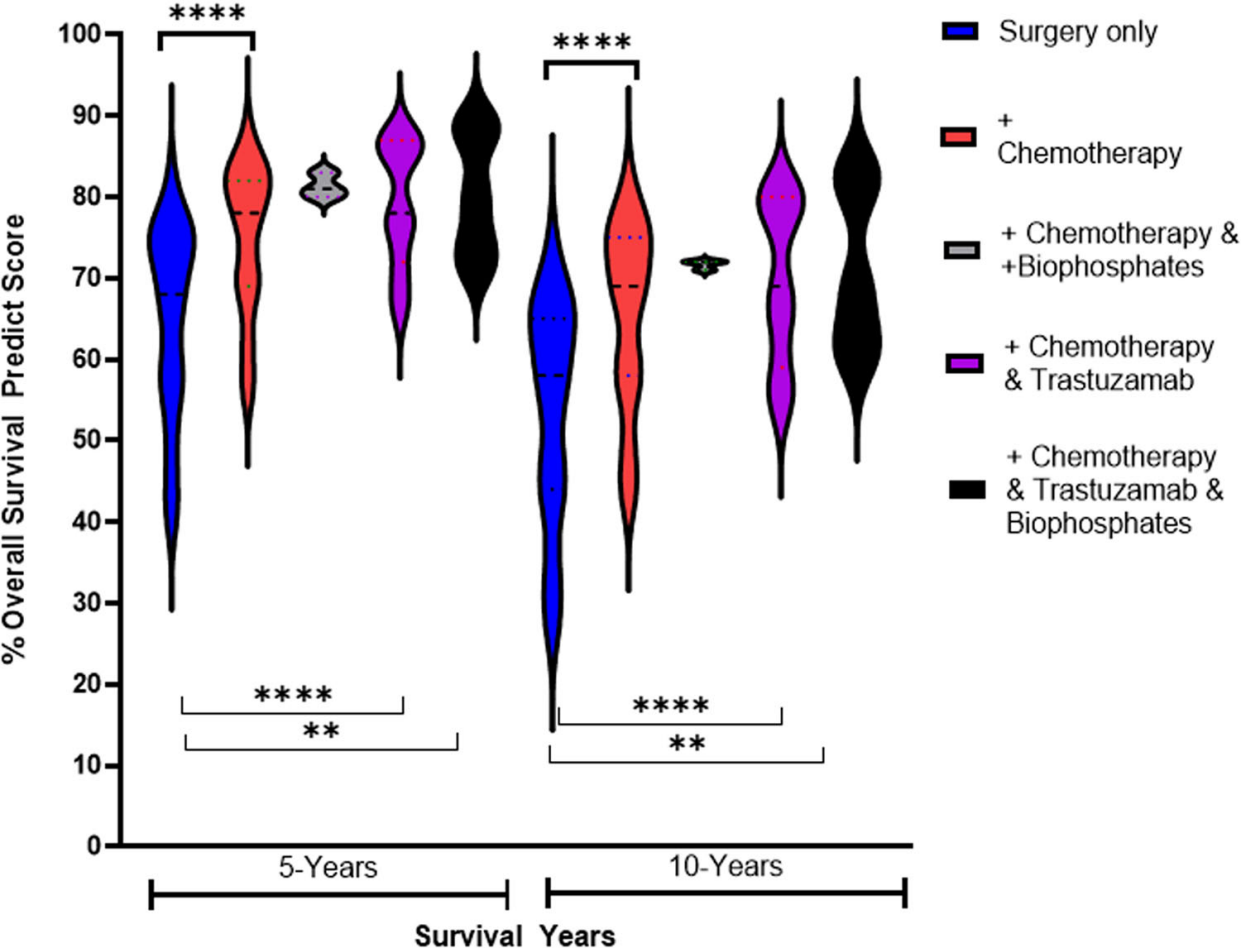
PREDICT Modelling of 5 and 10 Year Survival in ER^‐^ Breast Cancers Posttreatment.

The ER cohort (Table A2) had significantly increased OS with adjuvant chemotherapy at 5 and 10 years (*p* < 0.001), for women with HER2^+^ tumors, combining with trastuzumab (*p* < 0.001) and biophosphates for those post‐menopausal was beneficial (*p* = 0.002). The Wilcoxon signed rank test pairs shows that the observed difference were highly significant (*p* < 0.001) between both surgery and hormone therapy and surgery with adjuvant hormone and chemotherapy at 5 and 10 years. The Friedman test showed significant statistically significant difference between the means of these three groups (where the same subjects show up in each group, *n* = 119 (see Figure 4 for ER). The violin plots in Figure 4 illustrates how the distribution of survival in patients is improved by adding adjuvant treatment after surgery, across all added treatments, with the median high to low survival ranges decreasing from 30% (surgery) to just 2% (all adjuvants) at 5 years.

## 4 Discussion

This study aimed to improve the outlook of Iraqi women with breast cancer using personalised medicine strategies. First we implemented a large‐scale immunohistochemistry based tumor profiling study. The gold standard AJCC staging system considers biomarker testing with the hormone receptors ER, PR, and HER2 status, plus integration of Ki‐67 in the determination of the prognostic stage for breast cancer patients [29]. All four biomarkers have been reported in this study population according to the St Gallen consensus for predicting prognosis and treatment of patients with breast cancer [7, 8]. The tumor receptor profiles of each individual patient will require different treatment lines [30]. This requires accurate assessment of long‐term individual patient prognosis to guide clinical decision making and the use of prognostic tools.

The PREDICT platform has also been endorsed by the AJCC to show how treatments after surgery for breast cancer might improve survival rates, to help both the clinician and patient with the extremely challenging treatment decision making process. Therefore this study used PREDICT treatment modelling of delineated patient tumor profiles and clinicopathology parameters to show how different treatment scenarios can augment survival after surgery. An estimated 85.7% and 100% of ER^+^ and ER^‐^ patients were estimated to have a long‐term clinically relevant benefit when the right adjuvants are selected according to patient tumor profile using the platform. Undoubtable prognostic tools are a complementary strategy that can support countries such as Iraq that are facing ongoing healthcare resource limitations and cancer epidemics. However, a full clinical validation of the PREDICT platform with the prospective cohort is required to determine the accuracy of real‐life predictions in this ethnic population.

The immunohistochemistry investigations found that 81.8% of all cases express ER and 73.7% express PR, both biomarker levels match the literature ranges [31, 32, 33]. Although previously reported values in Iraq for ER and PR are lower at 66.8% and 64% respectively [5]. Both HER2 and PR were significantly associated with the histological type of tumor. The patients with breast cancer expressing ER are candidates for hormonal therapy either in adjuvant, neoadjuvant, metastatic, palliative, or even in preventive setting [34, 35]. [35]. In the adjuvant setting, the PREDICT platform indicated that administration of hormonal therapy would result in a clinically relevant 10‐year overall survival benefit of 5.45% compared to surgery alone.

Our study determined HER2 positivity in 20.8% of cases, this subset of patients are candidates for trastuzumab therapy. Predict modelling indicated significant clinical benefits of 12.54% and 16.02% with adjuvant trastuzumab combination therapy in both ER^+^ (*p* = 0.003) and ER^‐^ tumors (*p* < 0.001) respectively, compared to surgery alone.

The biomarker Ki‐67 was low in most cases (73.2%) and high in the remaining 26.8% of cases. Ki‐67 had significant clinicopathology association with LVI (*p* = 0.006), as previously reported by Kanyilmaz et al [36]. Tumors with LVI are also candidates for adjuvant therapies because of the high risk of recurrence and metastasis [37]. Furthermore Ki‐67 levels ≥ 25% are reported to have unfavorable outcomes in the literature [36]; this parameter was modelled using the PREDICT patient subset by selecting of Ki‐67 positive or negative options.

Ki‐67 coupled together with ER, PR and HER2 immunoexpression were used to delineate the prognostic molecular subtypes of breast cancer. Our classification of Iraqi breast cancer patients according to the St Gallen consensus determined luminal A (Luminal‐A‐like) and luminal B Luminal‐B‐like) as the most common types with a prevalence of 61.7% and 20.0% respectively, followed by HER2 enriched (HER2 positive) (9.5%) and basal‐like (TNBC) tumors (8.7%). Breast cancer‐specific survival is governed by subtype with shortest survival reported among HER2 + /ER‐ and basal‐like subtypes. Whereas the outlook of patients with Luminal A (Luminal‐A‐like) and Luminal B (Luminal‐B‐like) tumors is excellent with long‐term survival (5 years' survival rate is about 80–85%) with the advent of modern targeted treatments. An interesting finding of our study was the lowest rates of basal‐like (TNBC) breast cancer globally at just 8.7%. The basal molecular subtype is associated with high proliferation rate and gloomy clinical outcome [38]. This cancer subset has a high proliferative activity and strong invasiveness, causing poor relapse rate and a shortened overall survival [39]. In other countries such as Oman the distribution of basal subtypes reached 25.1% [26], and a high of 39% in African American women. Shomaf et al. found that Luminal A was the most common molecular type in Jordanian patent and represent 72% of cases and on other hand, basal like was the least common (14%) [15]. In a study by Alnegheimish et al. Saudi Arabia patients presented with luminal as the most common type (58.5) and HER2 positive as least common (44%). These differences may be attributed to racial and ethnic genetic differences.

The distribution of breast cancer molecular subtypes reported in Iraq and the noted variability with selected populations worldwide further highlight the urgent need for clinical validations of prognostic tools that are adapted to facilitate clinical decision making in this specific ethnic population. Further studies of the molecular profile of breast cancer in relation to clinical outcomes are fundamental to improving the management of patients with breast cancer, especially in different geographic regions worldwide. There are high unmet treatment needs for patients with the aggressive and chemo resistant basal subtype tumors.

### 4.1 Study Limitations

- The main study lacked molecular testing by PCR based or microarray gene expression for enhanced tumor profiling.
- Many cases were tru‐cut biopsy. Here, grading is not recommended as it may not reflect the actual grade and hence might over or under grade the tumor. Therefore, we couldn't include all cases analyzed in respect to grade against tumor markers as well as molecular or IHC groups.
- Tumor stage, LVI and lymph nodes involvement cannot be estimated from cases of tru‐cut biopsy. Therefore, we couldn't analyze all cases in respect to stage and lymph nodes involvement.

### 4.2 Future Directions

Collaborative international studies in breast cancer will help facilitate the introduction of personalized medicine therapies into routine clinical practise on a global scale for the benefit all races and ethnicities. The distribution of breast cancer subtypes is highly variable not only between races but across specific geographic populations. The prediction of tumor behaviour by identification of specific molecular characteristics and the downstream development of user‐friendly mathematical modelling tools is critical for improving tumor diagnostics and treatments and the survival of patients with cancer worldwide.

There are currently several sophisticated developments in the prediction of breast cancer outcomes using microarray and gene expression techniques. Examples of these techniques include MammaPrint (Agendia) and oncotype DX (Genomic Health) ‐ assays that enable outcome prediction using complex gene expression profiles [40]. For example, MammaPrint has a 70‐gene prognosis profile and was designed for selecting early (stage I‐II, ER+ or ER‐) breast cancer patients with low risk of developing metastasis, so that they could be spared adjuvant chemotherapy [41]. The assay classifies patient as high or low risk for metastasis depending on gene expression score [41]. Aggressive chemotherapy is provided for patient with high risk of metastasis while more conservative approach is applied for those with low score.

Furthermore, therapies directed to specific molecular targets of basal‐like tumors have rarely achieved clinically meaningful improvements in outcomes, and chemotherapy remains the standard of care. Therefore, understanding the mechanisms that drive drug resistance in patients with basal‐like breast tumors will be critical for designing novel therapies to prevent the development of metastatic disease and improve survival outcomes in this vulnerable population. Furthermore, systems based patient stratification tools are developed that investigate cell death pathways for the prediction of treatment responses, as reviewed by [42]. Modelling and data analytic tools are undoubtably the future cornerstones of personalised medicine, providing a comprehensive and meticulous view of the biological interactions between cellular components subjected to cancer drug treatments.

## Author Contributions

**Alaa Salah Jumaah:** conceptualization, investigation, writing–original draft, visualization, methodology, validation, software, formal analysis, project administration, data curation, supervision. **Roaa Ali Shaker:** methodology, writing–original draft. **Zainab Al‐Ali:** investigation, methodology, project administration, data curation. **Kaswer Musa Jaafar Altoriah:** methodology, software, writing–original draft. **Aseel Al‐Quzweni:** formal analysis. **Salam Salah Jumaah:** investigation, validation, writing–original draft, methodology. **Akeel Abed Yasseen:** project administration, supervision, resources. **Katherine Ann McAllister:** conceptualization, project administration, writing–original draft, writing–review and editing, visualization, formal analysis. **Hayder J Al Shiblawi:** investigation, supervision.

## Conflicts of Interest

The authors declare no conflicts of interest.

## Transparency Statement

The lead author Alaa Salah Jumaah affirms that this manuscript is an honest, accurate, and transparent account of the study being reported; that no important aspects of the study have been omitted; and that any discrepancies from the study as planned (and, if relevant, registered) have been explained.

## Supporting information

Supporting information.

## Data Availability

The data that support the findings of this study are available from the corresponding author upon reasonable request.
