# Supplementary material for: Immunohistochemical Breast Cancer Profiling Among Iraqi Women: Molecular Subtype Classification, Clinicopathology Associations, and Treatment‐Decision Making Tools: A Cross‐Sectional Study
Source: Health Sci Rep. 2025 May 19;8(5):e70553. doi: 10.1002/hsr2.70553 (PMC12086652; doi:10.1002/hsr2.70553)
Supplement: Supplementary file 1 — Supporting information. [file HSR2-8-e70553-s001.docx]

**Supporting information**

**Appendices**

**Table A1:**5-year and 10-year overall survival descriptive statistics returned by PREDICT platform script for all surgery and adjuvant treated patients stratified by ER+ status

|  | **Treatment**  **Modalities: surgery/ adjuvant** | | **Surgery** | | **Adjuvant Treatments** | | | | | | | | | | |
| --- | --- | --- | --- | --- | --- | --- | --- | --- | --- | --- | --- | --- | --- | --- | --- |
|  |  |  |  | | +Hormone | | + Hormone  + Chemo^a^ | | + Hormone  + Chemo  +BioP^b^ | | + Hormone  + Chemo  + Trast^c^ | +Hormone  +Chemo  +Trast  +BioP | | | |
|  | **Patients (n)** | | 119 | | 119 | | 119 | | 40 | | 9 | 2 | | | |
| **Predicted 5 Year OS (%)** | | | | | | | | | | | | | |  |  |
|  | Mean (SD) | | 90.08 (5.370) | | 92.52 (3.870) | | 94.57 (2.717) | | 94.05 (2.331) | | 95.78 (1.856) | 94.00 (1.414) | | | |
|  | Added adjuvant benefit (%) | | - | | 2.44 | | 4.49 | | 3.97 | | 5.7 | 3.92 | | | |
|  | Median | | 91.00 | | 93.00 | | 95.00 | | 94.00 | | 96.00 | 94.00 | | | |
| Range | | 30.00 | | 22.00 | | 15.00 | | 11.00 | | 6.000 | | | 2.000 | |  |
|  | P value, Wilcoxon matched-pairs signed rank, vs surgery | | | | <0.001  **** | | <0.001  **** | | <0.001  **** | | 0.003  ** | - | | | |
|  | Friedman Test, 3 groups (119 subjects) | | | | <0.001  **** | |  | | - | | - | - | | | |
|  | Friedman statistic | | 231.5 | |  | |  | | - | | - | - | | | |
|  | **Predicted 10 Year OS (%)** | | | | | | | | | | | | | | |
|  | Mean (SD) | | 76.13 (11.04) | | 81.58 (8.584) | | 86.12 (6.357) | | 84.48 (  5.435  ) | | 88.67  (4.359) | 83.50  (3.536) | | | |
|  | Added adjuvant benefit (%) | | - | | 5.45 | | 9.99 | | 8.35 | | 12.54 | 7.37 | | | |
|  | Adjuvant >5% benefit (number of cases %) | | | | 102 (85.7%) | | | | | | | | | | |
|  | Median | | 78.00 | | 83.00 | | 87.00 | | 85.00 | | 90.00 | 83.50 | | | |
|  | Range | | 59.00 | | 47.00 | | 35.00 | | 24.00 | | 14.00 | 5.000 | | | |
|  | P value, Wilcoxon matched-pairs signed rank vs surgery | | | | <0.001  **** | | <0.001  **** | | <0.001  **** | | 0.003  ** | - | | | |
|  | Friedman Test, 3 groups (119 subjects) | | | | <0.001  **** | |  | | - | | - | - | | | |
|  | Friedman statistic | | | | 238 | |  | | - | | - | - | | | |
|  |  |  |  |  |  |  |  |  |  |  |  |  |  |  |  |

^a^Chemo, chemotherapy; ^b^BioP, biophosphates; ^c^Trast, trastzumab

**Table A2:** 5 and 10-year overall survival descriptive statistics returned by PREDICT platform script for all surgery and adjuvant treated patients stratified by ER- status

|  | **Treatment**  **Modalities: Surgery/ Adjuvant** | | **Surgery** | **Adjuvant Treatments** | | | | | | | | | | | |
| --- | --- | --- | --- | --- | --- | --- | --- | --- | --- | --- | --- | --- | --- | --- | --- |
|  |  |  |  | +Chemo^a^ | | +Chemo  +BioP^b^ | | | + Chemo  + Trast^c^ | | | | +Chemo  +Trast  +BioP | | |
|  | **Patients (n)** | | 23 | 23 | | | 3 | | | 15 | | 10 | | | |
|  | **Predicted 5 Year OS (%)** | | | | | | | | | | | | | | |
|  | **Mean (SD)** | | 65.04  (11.95) | 75.09  (9.303) | | | 81.33  (1.528) | | | 79.13  (7.745) | | 80.40  (7.471) | | | |
|  | **Added adjuvant benefit (%)** | | - | 10.05 | | | 16.29 | | | 14.09 | | 15.36 | | | |
|  | **Median** | | 68.00 | 78.00 | | | 81.00 | | | 78.00 | | 80.00 | | | |
| **Median Range** | | 41.00                    32.00 | | | 3.00 | | | 21.00 | | |  | 18.00 | | |  |
|  | **P value, Wilcoxon matched-pairs signed rank, vs surgery** | | | <0.001  **** | | | 0.25  NS | | | <0.001  **** | | 0.002    ** | | | |
|  | **Predicted 10 Year OS (%)** | | | | | | | | | | | | | | |
|  | **Mean (SD)** | | 53.78  (13.82) | 65.48  (11.60) | | | 71.67  (0.5774) | | | 69.80  (10.16) | | 71.20  (9.875) | | | |
|  | **Added adjuvant benefit (%)** | | - | 11.7 | | | 17.89 | | | 16.02 | | 17.42 | | | |
|  | **Adjuvant >5% benefit (number of cases) n (%)** | |  | 23 (100%) | | | | | | | | | | | |
|  | **Median** | | 58.00 | 69.00 | | | 72.00 | | | 69.00 | | 69.00 | | | |
|  | **Range** | | 46.00 | 39.00 | | | 1.000 | | | 27.00 | | 24.00 | | | |
|  | **P value, Wilcoxon matched-pairs signed rank vs surgery** | | | <0.001  **** | | | 0.25  NS | | | <0.001  **** | | 0.002         ** | | | |
|  |  |  |  |  |  |  |  |  |  |  |  |  |  |  |  |

^a^Chemo, chemotherapy; ^b^BioP, biophosphates; ^c^Trast, trastzumab
